# Presidential address 2024: the expansion of computer-based testing to numerous health professions licensing examinations in Korea, preparation of computer-based practical tests, and adoption of the medical metaverse

**DOI:** 10.3352/jeehp.2024.21.2

**Published:** 2024-02-20

**Authors:** Hyunjoo Pai

**Affiliations:** President, Korea Health Personnel Licensing Examination Institute, Seoul, Korea; Hallym University, Korea


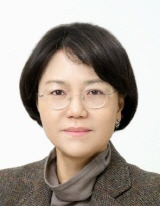


## Expansion of computer-based testing

Welcoming the hopeful new year, I will strive to meet expectations with a strong sense of responsibility for the changes and development of Korea’s health professions licensing examinations. In a previous presidential address, I mentioned improving item validity, adopting computer-based testing and clinical skills assessments in more health professions licensing examinations, and incorporating artificial intelligence and virtual reality in licensing examinations in Korea [1]. Out of these topics, the expansion of computer-based testing has been realized successfully. Furthermore, research is underway on preparing computerized adaptive testing and adopting the metaverse in licensing examinations; these research projects were selected by public offering, and the research results may be published soon. Commencing with the Korean Medical Licensing Examination in 2022, the Korea Health Personnel Licensing Examination has introduced and implemented computer-based testing across various healthcare professions. Successful computer-based testing administrations have been conducted in licensing examinations for dentists, doctors of oriental medicine, midwives, emergency medical technicians, health educators, oriental medicine pharmacists, medical technologists, and care workers, and the administration of these examinations has been stable to date.

Notably, the licensing examination for care workers underwent a significant transformation since its initial introduction in August 2010. After nearly 13 years, it shifted from the traditional paper-based exam format to a computer-based system, aligning with the digital transformation era. The care worker licensing examination was revamped into a year-round examination system, with the transition to computer-based testing on February 13, 2023. Since then, its presence has steadily solidified over the past year. With the introduction of this year-round examination system, candidates now have the flexibility to choose their preferred examination center, date, and time (morning/afternoon) for the examination. The entire process, from application submission to certificate of licensing issuance, has been streamlined into a computer-based one-stop system. Compared to the traditional written exam, it significantly reduces the time required from result announcements to certificate issuance. This system is more convenient for candidates and has affirmed the broader possibilities of computer-based testing.

The Korea Health Personnel Licensing Examination Institute intends to expand the implementation of computer-based testing further. In 2025, it plans to introduce computer-based licensing examinations for nurse assistants, first-level speech-language pathologists, and optometrists. Furthermore, in 2026, its goal is to extend computer-based testing to second-level speech-language pathologists, third-level health educators, pharmacists, and rehabilitation counselors. We hope to thoroughly prepare for the expansion of computer-based testing and anticipate a successful execution.

## Academic seminar on practical tests (clinical skill assessments) in the metaverse

In 2023, to commemorate the 31st anniversary of the establishment of the Korea Health Personnel Licensing Examination Institute, the institute organized an academic seminar with the theme of the development direction of national exam practical tests on May 16, 2023. The seminar comprised 2 sessions, addressing the current status and research findings on the computer-based practical tests and the fourth industrial revolution technologies and practical tests.

The implementation process and item development of computer-based practical tests were presented during the first session. Insights were drawn from practices on the American Board of Dental Examiners objective structured clinical examination (ADEX OSCE) in a presentation by Drs. Mark Armstrong and Stuart Blumenthal, CDCA-WREB-CITA. Furthermore, the development of an online OSCE (delivery and content) in Australia was presented by Professor Liz Farmer and Ms. Megan Lovett of the Australian Medical Council.

Drs. Mark Armstrong and Stuart Blumenthal outlined the ADEX OSCE, a tool for evaluating clinical judgment and decision-making in dentistry. Administered by the CDCA-WREB-CITA, the ADEX examinations assess the competence of dental and dental hygiene practitioners. The OSCE includes components such as patient treatment clinical examinations, endodontics, prosthodontics, vital tasks, and periodontal scaling. These examinations aim to ensure that dental professionals provide safe and competent care, embodying the principles of excellence, integrity, and fairness. The ADEX OSCE has evolved since 1969, adopting digital formats for improved security, exam quality, and candidate experience. The examination employs case-based scenarios to assess candidates’ abilities to apply knowledge, skills, and clinical judgment across various areas of dental practice. The design of exam content involves an occupational analysis every 5–7 years, ensuring the examination reflects current standards of dental care. Committees of experts continuously review and update the exam to maintain its relevance and integrity.

Professor Liz Farmer and Ms. Megan Lovett presented a comprehensive overview of the development and implementation of the Australian Medical Council’s online OSCE. They highlighted the adaptation to online delivery due to coronavirus disease 2019, leveraging Zoom to conduct clinical exams. The Australian Medical Council’s approach includes a detailed examination structure with 20 integrated stations, incorporating live patient simulations, examiner assessments, and a mix of clinical tasks. The transition to online exams faced challenges in content delivery, especially in areas like physical examination skills, but offered benefits such as accessibility and reduced costs for candidates. Continuous quality assurance and feedback have been crucial in refining the online examination process, with future enhancements focusing on improving interaction and content delivery and examining alternate ways to assess physical and procedural skills.

The second session dealt with topics including the use of standardized artificial intelligence patients in national exam practical tests (presented by Dr. Seong-Yong Kim, professor of oral and maxillofacial surgery at Chosun University) and the application of medical metaverse to the clinics and education (presented by Dr. Hyun-Young Kim, professor of pediatric surgery, Seoul National University).

Dr. Seong-Yong Kim discussed the use of artificial intelligence and virtual patients in medical and pharmacy education, highlighting the shift towards more interactive and effective methods of teaching communication skills. This presentation emphasized the importance of patient-centered communication and the challenges of traditional standardized patient training, such as cost and variability in performance. Virtual patients, as computer simulations, offer a cost-effective, standardized, and repeatable method for teaching and assessing communication and clinical skills. The presentation described the development of virtual patients using technologies like natural language processing, emotion modeling, and 3-dimensional animations, aiming to create realistic and engaging learning environments. This approach facilitates immersive learning experiences, allowing students to practice and build confidence in their communication, decision-making, and teamwork skills.

**Fig. 1.**
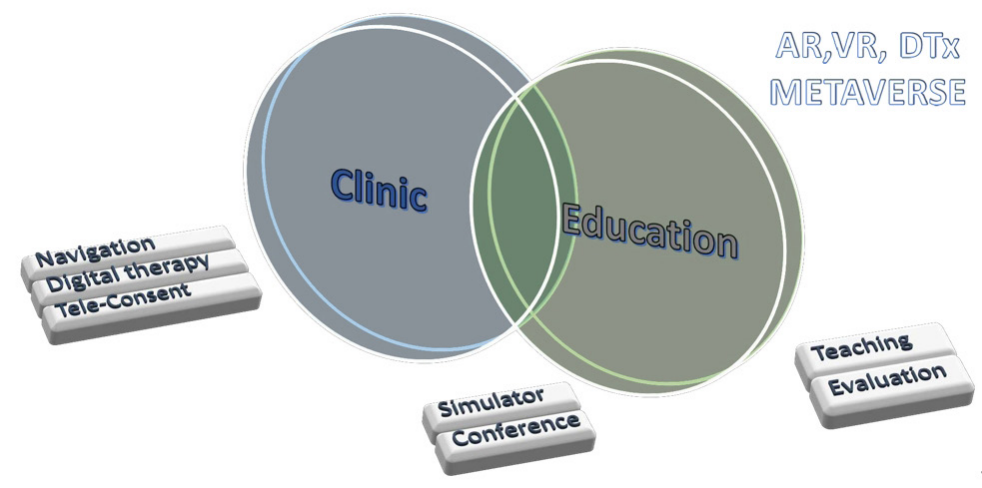
Application of the medical metaverse to the clinic and education. AR, augmented reality; VR, virtual reality; DT, digital therapy. Drawing by Dr. Hyun-Young Kim, professor of pediatric surgery, Seoul National University. CC-BY license.

Dr. Hyun-Young Kim explored the applications and implications of virtual reality, augmented reality, and the metaverse in medical fields, mainly focusing on education and clinical practice enhancements at Seoul National University Medicine and Hospital. The presentation delved into the integration of these technologies in creating immersive and interactive learning experiences, highlighting their potential to transform medical education and patient care (Fig. 1). Through examples such as surgery simulations and telemedicine, the text illustrates the convergence of digital medicine, artificial intelligence, and cloud technologies, suggesting a future where virtual and augmented realities significantly augment medical training and healthcare delivery. The document underscores the importance of advancing these technologies for better healthcare outcomes and preparing medical professionals for a digitally enhanced future. This information sharing and discussions facilitated a rich exchange of knowledge.

## Academic seminar on computerized adaptive testing in 2024

In 2024, the institute also plans to host an academic seminar with the theme of computerized adaptive testing from 10 AM to 5 PM on May 14, 2024 (Korea Standard Time) at KimKoo Museum & Library, located in Imjeong-ro 26, Yongsan, Seoul, Korea. Additionally, the 2024 academic seminar will be accessible for online participation through Zoom, and the presentation content will be translated in real-time, broadcasting in Korean and English. We look forward to the active participation of researchers who wish to share their knowledge and experiences related to computerized adaptive testing. The speakers and specific topics of the seminar and Zoom address will be announced again on the institute’s website (https://www.kuksiwon.or.kr/).

## Gratitude to the editorial board members and submitters

Throughout the past year, many changes and attempts have been made, such as the expansion of computer-based testing and the introduction of year-round testing. While these changes and initiatives have successfully taken root, continuous efforts are necessary to evolve in the ever-changing environment. I hope that *Journal of Educational Evaluation for Health Professions* (JEEHP) can be a driving force for such ongoing efforts by recruiting evidence-based data from all over the world.

Lastly, I express my gratitude to the dedicated editorial board members and staff of publishing companies who contribute to the development of JEEHP. I wish those who submit manuscripts to the journal and all those who visit the journal to be healthy and happy.
